# Cephalometric Assessment of Airway-Related Hyoid Position and Velar Morphology Across Skeletal Malocclusions: A Cross-Sectional Study

**DOI:** 10.3390/diagnostics16060947

**Published:** 2026-03-23

**Authors:** Gizem Yazdan Özen, Ali Kağan Özen, Nebiha Hilal Bilge

**Affiliations:** 1 Department of Orthodontics, Division of Clinical Sciences, Faculty of Dentistry, Kafkas University, Kars 36100, Türkiye; dtgzmyazdan@gmail.com; 2 Department of Oral and Maxillofacial Radiology, Division of Clinical Sciences, Faculty of Dentistry, Kafkas University, Kars 36100, Türkiye; n.hilalbilge@gmail.com

**Keywords:** hyoid bone, soft palate morphology, skeletal malocclusion

## Abstract

**Background:** The anatomical position of the hyoid bone and the morphological characteristics of the soft palate play a key role in upper airway patency, craniofacial balance, and the coordination of functional structures. These features may vary depending on skeletal pattern and gender. This study aimed to evaluate the relationship between hyoid bone position, soft palate morphology, skeletal classification, and gender using lateral cephalograms. **Methods:** A total of 120 individuals (60 females and 60 males) were classified as Skeletal Class I, II, or III based on the ANB (A Point–Nasion–B Point) angle. Measurements reflecting hyoid position and pharyngeal airway width were analyzed, including C3–H (distance from the third cervical vertebra to the hyoid bone), C3–RGn (distance from the third cervical vertebra to retrognathion), H–RGn (distance from the hyoid bone to retrognathion), PNS–UPW (distance from the posterior nasal spine to the upper pharyngeal wall), and U–MPW (distance from the uvula to the middle pharyngeal wall). Soft palate types were classified according to the You classification. Statistical analyses included ANOVA (analysis of variance), the Kruskal–Wallis test, the independent samples *t*-test, the Mann–Whitney U test, and the chi-square test. **Results:** Significant differences in C3–RGn, H–RGn, and U–MPW were observed between Skeletal Classes I–III and Classes II–III (*p* < 0.05). In contrast, C3–H and PNS–UPW did not differ significantly among skeletal classes. Soft palate types showed no significant association with skeletal classification or gender. Gender-based comparisons revealed significant differences in C3–H, C3–RGn, H–RGn, and PNS–UPW (*p* < 0.05). **Conclusions:** Mandibular-related hyoid measurements and the U–MPW parameter were associated with skeletal pattern, whereas C3–H and PNS–UPW remained relatively stable. Soft palate morphology was not significantly influenced by skeletal class or gender. These findings suggest that the hyoid–tongue–soft palate complex should be evaluated in conjunction with mandibular position during orthodontic diagnosis and treatment planning.

## 1. Introduction

The anatomical position of the hyoid bone and the physical characteristics of the soft palate are among the key structures influencing craniofacial balance, airway patency, and the functional integrity of these regions [1,2,3]. Variations in these areas may differ depending on individuals’ skeletal patterns, gender, and head posture [4,5]. In orthodontic treatment planning, the distinction between skeletal Classes I, II, and III is one of the fundamental parameters that guide diagnostic decisions and directly affect treatment strategies. Lateral cephalometric imaging is an indispensable diagnostic tool in orthodontic practice and is routinely obtained from every patient before treatment [6].

Although studies investigating the relationship between hyoid bone position or soft palate morphology and skeletal patterns are available in the literature, these structures have predominantly been evaluated separately or within specific patient groups [7,8,9]. The number of studies simultaneously examining the hyoid–soft palate complex together with mandibular-referenced measurements and pharyngeal airway parameters in a balanced, healthy adult population remains limited. In addition, systematic gender comparisons across skeletal classes have not been sufficiently addressed. Therefore, there is a need to support the existing body of knowledge with complementary data and to further refine it in order to achieve a more comprehensive understanding of the integrated relationship among the hyoid bone, soft palate, mandible, and airway structures. Our study provides a novel contribution to the literature by evaluating the hyoid–soft palate–airway complex in a balanced adult sample with equal gender distribution, integrating mandibular-referenced measurements together with the U–MPW parameter.

Awadalkreem et al. evaluated the hyoid bone position and soft palate morphology in skeletal Class I, II, and III patients using lateral cephalograms [4]. They reported significant differences in C3-H (the distance from the third cervical vertebra to the hyoid bone), C3-RGn (the distance from the third cervical vertebra to retrognathion), and H-RGn (the distance from the hyoid bone to retrognathion) measurements between Class II and Class III groups, with the greatest distances observed in Class III and the smallest in Class II. While leaf-shaped (Type I) soft palate was the most common in all classes, no significant association was found between soft palate morphology and gender or skeletal differences.

Ismail et al. evaluated the relationship between soft palate morphology and growth pattern, age groups, and gender in skeletal Class I, II, and III malocclusions using lateral cephalograms [2]. Skeletal classification was performed based on the ANB (A point–Nasion–B point angle), and growth pattern assessment included FMA (Frankfurt–Mandibular Plane Angle) and SNMP (Sella–Nasion–Mandibular Plane angle) measurements. Soft palate morphology was classified into six types according to the system defined by You et al. using lateral cephalometric radiographs. The findings indicated that the most common morphological type was the “leaf-shaped” (Type I) soft palate, which was reported as the predominant type across all skeletal classes. A statistically significant association was identified between soft palate morphology and skeletal classes. In contrast, no significant relationship was found between growth pattern and soft palate morphology. In terms of gender, a significant association between soft palate types and gender was reported. However, no statistically significant differences were observed between age groups and morphological variations.

In a study conducted by Šadzevičiūtė et al., hyoid bone position and pharyngeal airway characteristics were evaluated in different skeletal patterns (Classes I, II, and III) using lateral cephalograms [3]. Skeletal classification was based on the ANB (A point–Nasion–B point angle), and the distance from the hyoid bone to the third cervical vertebra was analyzed using the C3-H (the distance from the third cervical vertebra to the inferior border of the hyoid bone), while the distance to the mandibular symphysis was assessed using H-RGn (the distance from the inferior border of the hyoid bone to retrognathion). The results demonstrated that the distances from the hyoid bone to both C3 and the mandible differed significantly among skeletal classes. Specifically, in Class II individuals, the hyoid bone was positioned more posterior-superiorly, and the inferior airway space was significantly narrower. A positive correlation was identified between the inferior airway space and hyoid-related measurements; as the distance between the hyoid bone and the mandible or cervical vertebrae decreased, the inferior airway space also decreased. In contrast, no significant differences were found among groups in superior and middle airway measurements. Furthermore, mandibular length and the SNB (Sella–Nasion–B point angle) were reported to be associated with hyoid position. This study suggests that hyoid bone position is particularly related to mandibular sagittal position and may play a determining role in lower pharyngeal airway dimensions.

The position of the hyoid bone has been investigated in various studies. Muthukumar et al. evaluated the hyoid bone position relative to the mandible and the third cervical vertebra in skeletal Class I, II, and III malocclusions [10]. According to their findings, differences in C3-RGn and C3-H values were statistically significant between males and females in Class I malocclusion. In Class II, a significant difference was found between genders in the C3-H parameter, whereas no significant gender-related difference was observed in Class III. Additionally, positive correlations were identified between C3-RGn, C3-H, and H-RGn in Class I and III malocclusions.

Mortazavi et al. assessed hyoid bone position and gender differences across various skeletal patterns [11]. Although the C3-H distance was greater in males than females, it was lowest in Class II and highest in Class I individuals. Significant differences were found only between Class I and Class II. No significant differences were observed for H-RGn with respect to gender or skeletal class, although the lowest values occurred in Class II and the highest in Class III.

Studies on soft palate types are also present in the literature. Guttal et al. examined soft palate type, morphology, and dimensional variations across age and gender in an Indian subpopulation [12]. Type 1 was the most common in the general population and in both genders, with no significant correlation between age groups and soft palate types.

Kaurani and Suchita investigated the soft palate shapes in individuals classified by skeletal pattern according to the You classification [13]. They found that the most common soft palate shape was the crooked type (Type VI) in both the overall Marathwada population and in skeletal Class I and II groups, while the rat-tail type (Type VI) was most prevalent in skeletal Class III malocclusion.

The aim of our study is to examine the position of the hyoid bone and the morphology of the soft palate in individuals with skeletal Class I, II, and III using lateral cephalogram records, and to evaluate the relationship of these structures with gender and skeletal classification. The resulting data are expected to contribute to more comprehensive diagnostic assessments and individualized treatment planning in orthodontics.

## 2. Materials and Methods

This study was conducted by evaluating lateral cephalometric images taken for diagnostic purposes from individuals who presented with orthodontic problems to the Department of Orthodontics at Kafkas University Faculty of Dentistry. The research has a cross-sectional and descriptive design.

The study group consisted of individuals aged 18–30 years who had lateral cephalograms taken for diagnostic purposes. Inclusion criteria were completion of growth and development, absence of systemic disease, and possession of lateral cephalograms with adequate image quality. In addition, radiographically detectable pathological conditions that could affect airway measurements—such as marked adenoid hypertrophy, evident tonsillar hypertrophy, or abnormal anterior/posterior tongue positioning—were excluded from the study.

Individuals were classified into skeletal classes based on the ANB angle. Those with an ANB angle of 0–4° were categorized as Class I, those with >4° as Class II, and those with <0° as Class III. Each group included 20 females and 20 males, totaling 40 individuals per group; overall, 120 individuals were included in the study.

Lateral cephalometric images were obtained using a Ray-Scan (South Korea) digital imaging device equipped with panoramic and cephalometric attachments at the Department of Orthodontics, Kafkas University Faculty of Dentistry. All radiographs were taken in accordance with a standardized positioning protocol. During image acquisition, patients were positioned in an upright posture with the Frankfurt horizontal plane parallel to the floor, and efforts were made to achieve a natural head position as much as possible. The teeth were brought into maximum intercuspation, the lips were maintained at rest, and patients were instructed not to swallow and to keep the tongue in a passive position during exposure. Although ideal natural head position could not be guaranteed for all individuals due to the retrospective design, head posture was partially standardized. The obtained cephalometric films were evaluated using the web-based Odex Ceph AI Premium Edition (Ankara, Türkiye) analysis software. Various parameters representing the position of the hyoid bone and the morphology of the soft palate were analyzed.

All measurements were performed by an experienced orthodontist. To assess measurement reliability, repeat measurements were taken on 20 randomly selected cephalograms at a two-week interval, and intra-observer reliability was evaluated using the intraclass correlation coefficient (ICC). High agreement was achieved in repeated measurements, with an ICC value of 0.93.

Sample size analysis was performed using G*Power 3.1 software based on group means and standard deviations of the C3–RGn distance reported by Awadalkreem et al. in their study titled “Assessment of Hyoid Bone Position and Soft Palate Morphology in Different Skeletal Patterns Using Lateral Cephalograms: A Cross-sectional Study” [4] (Class I: 75.15 ± 8.36 mm, Class II: 71.13 ± 9.53 mm, Class III: 78.46 ± 5.40 mm). Using a 95% confidence interval and 90% power, the analysis indicated that at least 30 individuals were required in each group.

**Figure 1:**
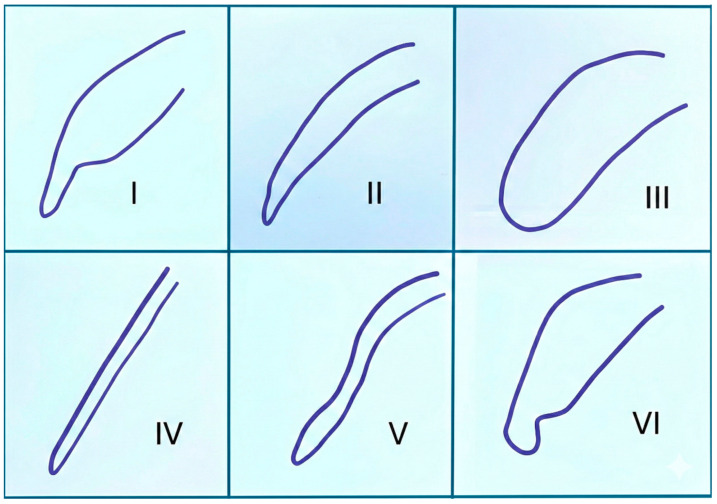
Type I—Leaf-shaped soft palate; Type II—Rat tail-shaped soft palate; Type III—Butt-like soft palate; Type IV—Straight line soft palate; Type V—S-shaped soft palate; and Type VI—Crook-shaped soft palate.

**Figure 2:**
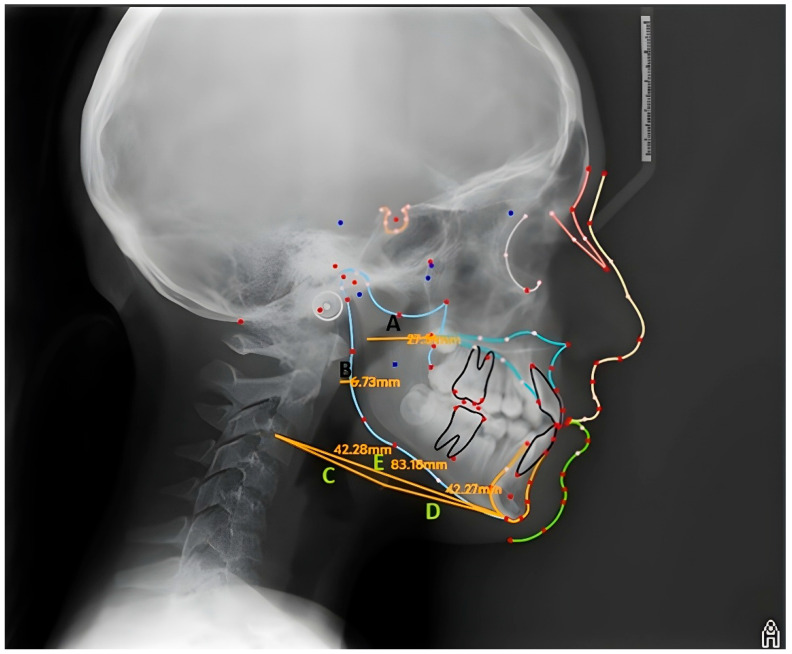
Lateral cephalometric measurements used (A: PNS-UPW, B: U-MPW, C: C3-H, D: H-RGn, and E: C3-RGn).

Morphological evaluation of the soft palate on lateral cephalometric radiographs was performed according to the six types defined by You et al. [14] (Figure 1). This classification is based on the contour shape of the soft palate on the lateral view, and each individual was matched with one of these types. In addition to this classification, quantitative analyses related to the spatial position of the hyoid bone and the width of the pharyngeal airway space were also conducted. All linear parameters used in the measurements and their descriptions are presented in detail in Table 1 and Figure 2.

All statistical analyses were performed using SPSS v25.0 software (SPSS, Inc., Chicago, IL, USA). In addition to descriptive statistics, the distribution of the data was evaluated using the Shapiro–Wilk test. Since the parameters used to assess hyoid bone position—C3-H, H-RGn, and PNS-UPW—showed normal distribution, one-way analysis of variance (ANOVA), post hoc tests with Bonferroni correction were applied for comparisons between skeletal Classes I, II, and III, as well as for comparisons among soft palate types. Independent samples *t*-test was used for gender-based comparisons of these parameters.

Because C3-RGn and U-MPW measurements did not conform to a normal distribution, the Kruskal–Wallis test was used to compare skeletal Classes I, II, and III, and soft palate types; pairwise comparisons between classes were performed using the Mann–Whitney U test. Gender-based comparisons of these parameters were also conducted using the Mann–Whitney U test. The chi-square test was used to compare the distribution of soft palate morphological types across skeletal classes and genders. A *p*-value of 0.05 was considered the threshold for statistical significance at 95% confidence intervals. Cohen’s d and Eta squared measurements were used for effect size.

## 3. Results

In this study, records of a total of 120 patients, including 60 males (50%) and 60 females (50%), were evaluated. The ages of the sample group ranged from 18 to 30 years. The mean age of the study population was 23.9 ± 3.6 years. The mean ages by group were calculated as follows: in Class I, 24.2 ± 3.4 years for females and 24.0 ± 3.6 years for males; in Class II, 23.5 ± 3.8 years for females and 23.9 ± 3.6 years for males; and in Class III, 23.7 ± 3.5 years for females and 23.9 ± 3.7 years for males.

When evaluating hyoid bone position across different skeletal classes, no significant difference was observed in the distribution of C3-H and PNS-UPW measurements among the classes. Significant differences were found between Classes I–III and II–III for C3-RGn, H-RGn, and U-MPW measurements (*p* < 0.05). This difference had a nearly strong effect size for the C3-RGn (η^2^ = 0.93), H-RGn (η^2^ = 0.74), and U-MPW (η^2^ = 0.85) parameters. No statistically significant association was observed between hyoid measurements and the distribution of soft palate types.

Significant differences were found between genders in the distribution of the C3-H, C3-RGn, H-RGn, and PNS-UPW parameters (*p* < 0.05). This difference had a small effect size for all parameters (C3-H (d = 0.4), C3-RGn (d = 0.3), H-RGn (d = 0.4), and PNS-UPW (d = 0.3)).

The distributions of hyoid position parameters and pharyngeal wall-related measurements across skeletal classes and genders are presented in Table 2 and Table 3.

**Figure 3:**
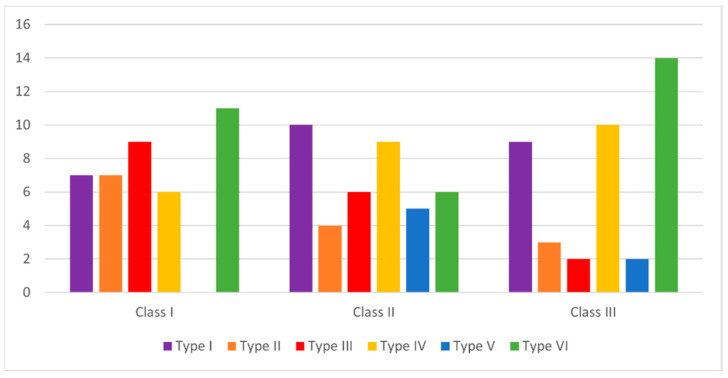
The prevalence of the soft palate shapes among the different skeletal classes.

No statistically significant difference was found in the distribution of soft palate types between genders or among skeletal classes. The most common soft palate type in females was Type IV (25%), whereas in males the most common type was Type VI (33.3%). Overall, Type VI was the most frequently observed soft palate morphology (25.8%). The most common soft palate shape in Class I was Type VI (27.5%), in Class II it was Type I (25%), and in Class III it was Type VI (35%) (Figure 3).

## 4. Discussion

In this study, the hyoid position and soft palate morphology of 120 individuals aged 18–30 years were examined according to skeletal classes and gender. While no significant differences were found in C3-H and PNS-UPW measurements among skeletal classes, C3-RGn, H-RGn, and U-MPW values showed significant differences between Classes I–III and Classes II–III (*p* < 0.05). No significant association was found between the distribution of soft palate types and skeletal class or gender. However, C3-H, C3-RGn, H-RGn, and PNS-UPW measurements differed significantly between genders (*p* < 0.05).

Awadalkreem et al. reported that hyoid bone position and soft palate morphology vary across skeletal classes [4]. They found significant differences in C3-H, C3-RGn, and H-RGn measurements between Class I and Classes II–III, emphasizing increased distances particularly in Class III individuals. They also reported that the leaf-shaped palate was most common, whereas the hook-shaped palate was least common. In our study, findings for C3-RGn and H-RGn were consistent with those of Awadalkreem et al., but no significant class-related difference was found for C3-H. Similarly, we found significant class differences in U-MPW, whereas Awadalkreem et al. did not report this parameter. Regarding soft palate types, although the general distribution pattern was similar between the two studies, no statistically significant relationship was observed in our sample. These discrepancies may stem from differences in sample size, age range, measurement parameters, and software-assisted analysis. Additionally, the two-dimensional nature of cephalometric imaging and variations in head–neck posture may also have influenced the results.

In the study by Ismail et al., the most frequently observed soft palate morphology was Type 1 (Leaf-shaped), which was reported as the predominant type across all skeletal classes (Class I: 42.4%, Class II: 42%, Class III: 57.7%) [2]. A statistically significant association was identified between soft palate morphology and skeletal class (*p* < 0.05). Furthermore, a significant relationship was found between morphological type distribution and gender (*p* < 0.05); Type 1 was reported as the most common type in both males (45.8%) and females (42.3%). In contrast, our study did not demonstrate a statistically significant association between soft palate morphology and skeletal class. Type VI was the most frequently observed morphology in Class I (27.5%) and Class III (35%) individuals, whereas Type I (25%) was most common in Class II. Overall, Type VI (25.8%) was the most prevalent morphology in our sample. With respect to gender, Type IV (25%) was most common in females, while Type VI (33.3%) was most frequent in males; however, no statistically significant association was found between soft palate morphology and gender. These differences in statistical significance may be attributed to variations in skeletal class distribution within the sample, differences in the evaluation of growth patterns, and methodological differences in gender distribution. Additionally, the lack of quantitative control for functional factors such as mouth-breathing history, adenoid hypertrophy, and other airway-related variables may have influenced morphological distribution. Moreover, the two-dimensional nature of lateral cephalograms and the reliance on visual morphological classification may also have contributed to inter-study variability.

In the study by Šadzevičiūtė et al., the distance from the hyoid bone to the third cervical vertebra and to the mandibular symphysis was reported to be greatest in Class III individuals and lowest in Class II, with these differences being statistically significant (*p* < 0.05) [3]. Similarly, the inferior airway space was found to be widest in Class III and narrowest in Class II, also demonstrating statistical significance. In that study, the GoB1–R3 and P–R2 parameters were used to represent lower airway space; among these, the P–R2 measurement—representing the distance from the posterior soft palate region to the posterior pharyngeal wall—is anatomically and methodologically comparable to the U–MPW (uvula tip–middle pharyngeal wall) parameter used in our study and may therefore be interpreted as an equivalent measure. Consistent with these findings, our study demonstrated higher U–MPW values in Class III and lower values in Class II, with statistically significant differences between Class I–III and II–III (*p* < 0.05). This parallels the tendency observed in the other study, where inferior airway width increased in Class III and decreased in Class II as indicated by the P–R2 parameter. Likewise, mandibular-referenced hyoid measurements (C3-RGn and H-RGn) were reported to be higher in Class III in both studies. However, in our study, no significant differences were observed among skeletal classes for C3-H and PNS-UPW. Regarding gender, while no significant intergroup differences were reported in the other study, our findings revealed statistically significant differences between males and females in C3-H, C3-RGn, H-RGn, and PNS-UPW (*p* < 0.05). Overall, both studies support the tendency toward a more anteriorly positioned hyoid bone and a wider lower/oropharyngeal airway in Class III individuals. The observed differences may be related to variations in sample age (mean age of 14 years in the other study versus a young adult population in our study), skeletal class distribution, and methodological differences in measurement parameters.

The relationship of the hyoid bone and soft palate with the upper airway has been widely investigated in different skeletal patterns and airway anomalies [7,15]. Our findings, particularly the significant differences in mandibular-referenced measurements such as C3-RGn and H-RGn in skeletal Class III individuals, support the influence of mandibular position on the anteroposterior placement of the hyoid bone [4]. The absence of class differences in PNS-UPW suggests that sagittal dimensions of the nasopharyngeal region may not be decisive in distinguishing skeletal Class I–III relationships.

The hyoid bone functions as part of an integrated unit with the tongue, suprahyoid/infrahyoid muscles, and the mandible [16]. In Class III individuals, anterior positioning of the mandible may increase the relative anterior placement of the hyoid bone with respect to RGn, which could explain the C3-RGn and H-RGn differences. The variation observed in U-MPW indicates that the assessment of the oropharyngeal lumen at the uvula level may be influenced by mandibular or lingual posture. In contrast, the lack of significant changes in PNS-UPW may be due to PNS being a maxillary-based reference point and the possibility that maxillary involvement in Class I and Class III discrepancies may not have been sufficiently represented. We believe that if skeletal anomalies were subgrouped as maxillary-based, mandibular-based, or bimaxillary, significant differences in PNS-UPW might also emerge.

The absence of significant associations between soft palate types and skeletal classes may be attributed to the fact that the You classification is morphology-based and does not directly reflect functional characteristics (e.g., muscle tone, velopharyngeal closure patterns) [14]. Gender-related differences are consistent with known dimorphisms in cranial base dimensions, soft tissue thickness, and pharyngeal volumes [5]. Furthermore, the lack of association between soft palate type and skeletal class may also be influenced by performing measurements in 2D projection, momentary tongue–soft palate interactions, and variations in head–cervical posture [17].

Radiographic assessment of hyoid bone position and soft palate morphology using lateral cephalograms may provide valuable insight for diagnosing and managing different skeletal patterns. Class-related differences in mandibular-referenced measurements (C3-RGn, H-RGn) highlight the need to consider mandibular position in functional airway evaluations [18]. Differences in U-MPW, especially in Class III individuals, may indicate the need for functional tests assessing tongue–soft palate relationships (e.g., nasal/oral breathing habits, snoring history) and possibly 3D imaging. Gender differences suggest that gender should be considered during pre-treatment evaluation, especially for assessing the tendency for retrotongual narrowing [19]. The absence of differences in PNS-UPW indicates that assessing the nasopharyngeal region in a single 2D section may be insufficient; CBCT-based volumetric measurements or dynamic methods (endoscopy and phonation-stage imaging) may be required when indicated [20].

In this study, the ANB angle was used to determine skeletal classification. The ANB angle is one of the most widely used parameters in orthodontic literature for evaluating sagittal skeletal relationships and is accepted as a clinical standard. Its high comparability with other studies and its routine use in clinical practice were influential in its selection. Furthermore, since the primary aim of our study was to compare the relationship between the hyoid bone and the soft palate across sagittal skeletal classes, classification was intended to be based on a homogeneous, reproducible criterion consistent with the literature. Nevertheless, it is well known that the ANB angle may be influenced by factors such as cranial base length, Nasion position, and vertical growth pattern, and therefore possesses inherent geometric limitations [21,22]. The exclusion of additional sagittal and vertical indicators such as Wits appraisal, SN-MP, or FMA should be considered a methodological limitation of this study. Future studies incorporating multiple skeletal parameters may allow for more detailed subgroup analyses and a more comprehensive interpretation of the findings.

One of the major limitations of this study is the inability to fully control certain potential confounding variables related to airway assessment. Variables such as BMI, breathing pattern, craniocervical angle, degree of tonsillar hypertrophy, and dynamic functional factors could not be quantitatively evaluated due to the retrospective nature of the data. Although radiographs were obtained according to a standardized protocol and head posture as well as radiographically assessable factors were partially standardized, the potential influence of these variables on the measurements cannot be completely excluded [23,24]. Obtaining cephalograms in a strict natural head position might have increased reliability. Therefore, the findings should be interpreted with caution. In future prospective studies, objective evaluation of these variables may contribute to a more comprehensive interpretation of the results. Additionally, the creation of age subgroups and the assessment of age-related differences could have enhanced clinical relevance. The single-center and regionally limited sample also restricts the generalizability of the findings.

In this study, lateral cephalometric imaging was utilized, and the methodological limitations inherent to two-dimensional (2D) projection in airway assessment should be considered [25]. Lateral cephalograms do not reflect volumetric characteristics or transverse dimensions of the airway; they allow only linear measurements in the sagittal plane. Moreover, dynamic properties of soft tissues, respiratory phase, tongue posture, and functional variability cannot be evaluated using this method [26,27]. Superimposition effects and projection distortion may also affect measurement accuracy and interpretation [28]. Therefore, the obtained findings do not directly represent airway volume but rather reflect morphological and linear structural relationships in the sagittal plane. In clinical interpretation, the results should not be considered as three-dimensional volumetric analyses but should instead be evaluated within the framework of structural relationships. Nevertheless, lateral cephalometry remains a routine component of orthodontic practice and continues to be widely used in clinical evaluation due to its low radiation dose, accessibility, and standardization advantages [29]. However, in cases where advanced airway analysis is required, three-dimensional imaging modalities such as CBCT may provide a more comprehensive assessment [30].

Future studies should integrate CBCT-based 3D volumetric analyses, functional assessments such as rhinomanometry or PSG, and craniocervical posture evaluations through prospective and multicenter designs. To better understand the clinical relevance of soft palate types, dynamic evaluations (phonation and swallowing) and simultaneous analysis of tongue posture are recommended. Longitudinal investigations on the effects of orthodontic and/or orthognathic treatments on the hyoid–airway complex are also needed.

## 5. Conclusions

In conclusion, mandibular-referenced hyoid measurements (C3-RGn and H-RGn) and the U-MPW parameter demonstrated statistically significant differences among skeletal classes, whereas C3-H and PNS-UPW measurements showed similar distributions. No significant association was identified between soft palate morphotypes and skeletal class or gender. These findings suggest a possible relationship between skeletal pattern and hyoid–airway parameters. However, due to the cross-sectional design of the study, these associations cannot be interpreted as causal. The results may contribute to increased clinical awareness regarding the evaluation of the hyoid–tongue–soft palate complex in conjunction with mandibular position during orthodontic diagnosis and treatment planning.

## Figures and Tables

**Table 1:** Definitions of the parameters used.

Abbreviations	Definition
C3-H	The distance from the third cervical vertebra (C3) to the inferior border of the hyoid bone
C3-RGn	The distance from the third cervical vertebra to retrognathion (the most posterior point of the mandibular symphysis)
H-RGn	The distance from the inferior border of the hyoid bone to retrognathion
PNS-UPW	The distance between PNS (Posterior Nasal Spine) and UPW (Upper Pharyngeal Wall)
U-MPW	The distance between U (tip of the uvula) and MPW (Middle Pharyngeal Wall)

**Table 2:** Distribution of hyoid position and pharyngeal wall-related parameters among skeletal classes.

	C3-H			C3-RGn			H-RGn			PNS-UPW			U-MPW		
Class	Mean ± Std	Min	Max	Mean ± Std	Min	Max	Mean ± Std	Min	Max	Mean ± Std	Min	Max	Mean ± Std	Min	Max
I	33.89 ± 5.31	20.91	46.22	72.31 ± 9.75	44.76	93.69	42.17 ± 6.67	26.86	58.44	21.40 ± 3.62	12.92	30.28	9.13 ± 2.86	5.36	16.30
II	33.58 ± 3.95	25.28	42.30	72.34 ± 7.11	55.58	87.87	42.70 ± 5.18	29.61	49.91	22.11 ± 4.14	11.04	29.98	8.92 ± 2.09	4.58	15.19
III	34.14 ± 4.25	24.47	43.09	78.43 ± 10.20	59.50	96.86	46.42 ± 8.20	31.09	69.94	21.63 ± 4.33	13.21	29.17	10.70 ± 3.06	4.28	16.96
95%	*p* = 0.85			I–III: *p* = 0.01 II–III: *p* = 0.01			I–III: *p* = 0.01 II–III: *p* = 0.04			*p* = 0.72			I–III: *p* = 0.02 II–III: *p* = 0.01		

**Table 3:** Distribution of hyoid position and pharyngeal wall-related parameters between genders.

	C3-H			C3-RGn			H-RGn			PNS-UPW			U-MPW		
Gender	Mean ± Std	Min	Max	Mean ± Std	Min	Max	Mean ± Std	Min	Max	Mean ± Std	Min	Max	Mean ± Std	Min	Max
Female	32.95 ± 3.97	20.91	41.42	76.15 ± 8.56	44.76	96.59	45.23 ± 6.15	26.86	62.12	22.49 ± 3.84	11.04	30.28	9.80 ± 2.81	5.49	16.86
Male	34.79 ± 4.84	24.47	46.22	72.56 ± 10.10	55.41	96.86	42.29 ± 7.53	29.94	69.94	20.93 ± 4.08	13.21	29.17	9.40 ± 2.81	4.28	16.30
95%	*p* = 0.02			*p* = 0.01			*p* = 0.02			*p* = 0.03			*p* = 0.42		

## Data Availability

The datasets generated and/or analyzed during the current study are not publicly available.

## Abbreviations

The following abbreviations are used in this manuscript:

ANB	A Point–Nasion–B Point Angle
CBCT	Cone Beam Computed Tomography
C3	Third Cervical Vertebra
C3-H	Distance from Third Cervical Vertebra to Hyoid Bone
C3-RGn	Distance from Third Cervical Vertebra to Retrognathion
H-RGn	Distance from Hyoid Bone to Retrognathion
ICC	Intraclass Correlation Coefficient
MPW	Middle Pharyngeal Wall
PNS	Posterior Nasal Spine
PNS-UPW	Distance from Posterior Nasal Spine to Upper Pharyngeal Wall
PSG	Polysomnography
RGn	Retrognathion
SPSS	Statistical Package for the Social Sciences
U	Tip of the Uvula
U-MPW	Distance from Uvula to Middle Pharyngeal Wall
UPW	Upper Pharyngeal Wall
